# Early Exchange Transfusion in Critical Pertussis: A Case Report Supporting Timely Intervention

**DOI:** 10.1155/crpe/7016973

**Published:** 2026-09-04

**Authors:** Eiman Al-Hashemi, Osama Shalaby, Fajer Altammar

**Affiliations:** ^1^ Pediatric Intensive Care Unit, Department of Pediatrics, New Jahra Hospital, Ministry of Health, Jahra, Kuwait, behdasht.gov.ir; ^2^ Faculty of Pediatrics, Kuwait Institute for Medical Specialization, Kuwait, Kuwait

## Abstract

**Background:**

Critical pertussis is a rapidly progressive, life‐threatening illness in infants, often presenting with hyperleukocytosis, respiratory failure, and hemodynamic instability. Mortality remains high despite supportive advances, and no standardized guidelines exist to guide the use of exchange transfusion in this setting.

**Objective:**

To describe the outcomes of early exchange transfusion in infants with critical pertussis managed in a high‐acuity tertiary PICU in Kuwait and to advocate for the development of a standardized national protocol.

**Methods:**

This prospective case report includes three infants with PCR‐confirmed *Bordetella pertussis* infection admitted between January and December 2024 to the Pediatric Intensive Care Unit (PICU) of New Jahra Hospital, Kuwait’s busiest tertiary‐level center. The decision to initiate exchange transfusion was based on consensus clinical assessment integrating clinical status and laboratory progression. Once the decision to proceed was made, double‐volume exchange transfusion was performed according to a unit‐specific procedural protocol developed within our institution. Clinical data, laboratory trends, and short‐term outcomes were reviewed.

**Results:**

All infants presented with severe respiratory failure, marked hyperleukocytosis (> 30–60 × 10^9^/L), and evolving hemodynamic instability. Exchange transfusion performed within 2–4 days of admission led to prompt leukoreduction, improved perfusion, and clinical stabilization. All survived to discharge and remained well at 6‐week follow‐up. Thrombocytopenia occurred following exchange transfusion and resolved with supportive care. Seizures and iatrogenic opioid withdrawal occurred during the PICU course in Cases 1 and 2 and were not considered complications directly attributable to exchange transfusion. National feedback from PICU unit heads highlighted broad support for protocol standardization.

**Conclusion:**

Early exchange transfusion, guided by evolving clinical severity and laboratory trends, may contribute to favorable outcomes in infants with critical pertussis when considered before refractory pulmonary hypertension develops. These cases support timely recognition and intervention while underscoring the need for standardized national practice, maternal Tdap immunization, and strengthened disease surveillance.

## 1. Introduction

Pertussis, or whooping cough, caused by *Bordetella pertussis*, can lead to critical illness in young infants, especially those under three months of age [1, 2]. Characterized by hyperleukocytosis, respiratory failure, and multiorgan dysfunction, critical pertussis has a mortality rate ranging from 10% to 30% [1, 2]. Standard treatment is largely supportive [3]. However, the rapid progression and poor prognosis have led to the consideration of alternative therapies, such as exchange transfusion [1, 4–6]. This case report describes the implementation of early exchange transfusion as a treatment modality for critical pertussis and proposes the establishment of a national protocol.

## 2. Methods

This was a prospective case report conducted at the Pediatric Intensive Care Unit (PICU) of New Jahra Hospital, the highest‐volume tertiary PICU in Kuwait, admitting approximately 700 patients annually. Three infants admitted with critical pertussis between January and December 2024 were included. Diagnosis was confirmed via *Bordetella pertussis* PCR. Critical pertussis was defined as severe disease in infants and young children associated with one or more of the following: marked hyperleukocytosis (often > 30–50 × 10^9^/L), hypoxemic respiratory failure requiring advanced ventilatory support, hemodynamic instability, and/or evidence of evolving pulmonary hypertension.

We adopted a consensus‐based decision‐making approach to determine when exchange transfusion should be considered. This process integrated multiple predefined trigger thresholds with the patient’s clinical status and biochemical progression. Key triggers included a rising white blood cell count exceeding 30 × 10^9^/L in the context of respiratory compromise, an increasing neutrophil‐to‐lymphocyte ratio, and/or unexplained tachycardia. Once the decision to proceed was made, double‐volume exchange transfusion was performed according to a unit‐specific procedural protocol developed within our institution. This procedural protocol standardized how the exchange transfusion was performed; it did not determine the indication or timing for initiating exchange transfusion. At the time of this report, the procedural protocol remained under final institutional review and is, therefore, not reproduced in full here.

## 3. Results

Patient characteristics, therapies, exchange transfusion details, and outcomes are summarized in Table 1.

**TABLE 1:** Summary of patient characteristics, therapies, exchange transfusion details, and outcomes.

	Case 1	Case 2	Case 3
Age	5 months and 24 days	37 days	3 months and 1 day
Risk factors: Prematurity	Yes (34 weeks)	No (37 weeks)	No (39 weeks)
Vaccine status	Unvaccinated	Unvaccinated	Unvaccinated
Comorbidities	Prematurity	None	Neonatal hypothyroidism
Maternal immunization	Unknown	No	Unknown
Symptoms: Paroxysmal cough	No	Yes	Yes
Cough > 7 days	No	Yes	Yes
Cyanosis	Yes	Yes	Yes
Fever (*T* > 38)	Yes	No	Yes
Inspiratory whoop	No	No	No
Decreased feeding	Yes	Yes	Yes
Days from initial symptoms to PICU admission	5	5	4
Reason for PICU admission	Respiratory distress, hyperleukocytosis	Respiratory distress, hypoxia, hyperleukocytosis	Respiratory distress, hypoxia, hyperleukocytosis
Exchange transfusion day	Day 3	Day 2	Day 4
Type of exchange transfusion	Double × 2	Double	Double
Peak WBC (10^9^/L)	61.6	30.7	42.7
Peak PLT (10^9^/L)	668	531	678
Pre exchange WBC (10^9^/L)	61.64	30.2	35.16
Post exchange WBC (10^9^/L)	28	20.1	24.24
Neutrophil/lymphocyte (N/L) ratio	0.72	1.07	0.62
Pre exchange HR	185	175	170
Post exchange HR	140	114	140
Pre exchange BP	90/35	64/35	70/32
Post exchange BP	85/40	60/30	65/35
Echo findings prior to exchange	Normal (no pulmonary HTN)	PFO (no pulmonary HTN)	Normal (no pulmonary HTN)
S/F ratio prior to exchange	165	300	222
Type of respiratory support	IMV, HFOV, iNO	IMV, HFOV	IMV, HFOV
IMV days	16	9	18
NIV days	4	1	2
Vasoactives/inotropes	None	Epinephrine	None
Hyperhydration	No	No	No
Steroids	No	No	No
Multiorgan failure	No	No	No
Complications	Seizures, thrombocytopenia, withdrawal	Seizures, thrombocytopenia, withdrawal	Thrombocytopenia
PICU LOS (days)	26	19	22
Outcome	Recovery	Recovery	Recovery

*Note:* Withdrawal in Cases 1 and 2 refers to iatrogenic opioid withdrawal during the PICU course and was not considered a complication directly attributable to exchange transfusion.

Abbreviations: HFOV = high‐frequency oscillatory ventilation, HTN = hypertension, IMV = invasive mechanical ventilation, iNO = inhaled nitric oxide, NIV = noninvasive ventilation.

### 3.1. Case 1

A 5‐month‐and‐24‐day‐old male infant, born prematurely at 34 weeks’ gestation, presented with cyanosis, fever, and decreased feeding. He was unvaccinated, with maternal vaccination status unknown. On admission, he demonstrated respiratory distress, hyperleukocytosis, and hemodynamic instability. Peak WBC count reached 61.6 × 10^9^/L with a neutrophil‐to‐lymphocyte ratio of 0.72. Echocardiography excluded pulmonary hypertension. Within 72 h of admission, the infant required invasive mechanical ventilation, high‐frequency oscillatory ventilation (HFOV), and inhaled nitric oxide (iNO). On Day 3, a double‐volume exchange transfusion was performed. A second exchange transfusion was undertaken after the WBC count rebounded to approximately 25 × 10^9^/L, accompanied by persistent/worsening clinical findings. Following the exchange procedures, the WBC count was 28 × 10^9^/L and heart rate decreased from 185 to 140 bpm. Blood pressure remained stable (90/35 ⟶ 85/40 mmHg). Thrombocytopenia occurred following exchange transfusion and was managed with supportive care. Seizures and iatrogenic opioid withdrawal also occurred during the PICU course but were not considered complications directly attributable to exchange transfusion. The infant required 16 days of mechanical ventilation and 4 days of noninvasive ventilation (NIV), with a total PICU stay of 26 days. He was discharged in stable condition with good recovery at follow‐up.

### 3.2. Case 2

A 37‐day‐old term infant presented with paroxysmal cough, cyanosis, hypoxia, and poor feeding. He was unvaccinated, with no comorbidities, and maternal vaccination was not received. At admission, peak WBC count was 30.7 × 10^9^/L with a neutrophil‐to‐lymphocyte ratio of 1.07. The infant rapidly progressed to respiratory failure requiring intubation, invasive ventilation, and HFOV. On Day 2 of admission, a double‐volume exchange transfusion was performed, lowering WBC count from 30.2 × 10^9^/L to 20.1 × 10^9^/L and reducing heart rate from 175 to 114 bpm. Blood pressure remained relatively unchanged (64/35 ⟶ 60/30 mmHg). Echocardiography revealed a patent foramen ovale without pulmonary hypertension. The infant required epinephrine support for hemodynamic instability. Thrombocytopenia occurred following exchange transfusion and resolved with supportive management. Seizures and iatrogenic opioid withdrawal also occurred during the PICU course but were not considered complications directly attributable to exchange transfusion. He required 9 days of invasive ventilation and 1 day of NIV, with a total PICU stay of 19 days, and was discharged in stable condition with full recovery and good neurodevelopmental outcome at follow‐up.

### 3.3. Case 3

A 3‐month‐and‐1‐day‐old term infant with a past history of neonatal hypothyroidism presented with cough, cyanosis, and decreased feeding. He was unvaccinated, with maternal immunization status unknown. On admission, peak WBC was 42.7 × 10^9^/L with a neutrophil‐to‐lymphocyte ratio of 0.62. The infant developed respiratory failure requiring invasive ventilation and HFOV. On Day 4 of admission, a double‐volume exchange transfusion was performed, reducing WBC count from 35.2 × 10^9^/L to 24.2 × 10^9^/L, and heart rate from 170 to 140 bpm. Blood pressure remained stable (70/32 ⟶ 65/35 mmHg). Echocardiography was normal, without evidence of pulmonary hypertension. Complications included thrombocytopenia, which resolved with supportive care. The infant required 18 days of invasive ventilation and 2 days of NIV, with a PICU stay of 22 days. He was discharged in stable condition with good neurodevelopmental outcome at follow‐up.

## 4. Discussion

This case report is timely in light of the global resurgence of pertussis, including in countries with longstanding immunization programs. Despite widespread adoption of the acellular pertussis (aP) vaccine, which replaced the whole‐cell vaccine in many regions in the 1990s due to improved safety profiles, a notable increase in pertussis incidence has been observed over the past decade. Waning immunity, antigenic variation in circulating *Bordetella pertussis* strains, and reduced long‐term vaccine effectiveness are increasingly recognized contributors to this trend [7–10].

Infants—particularly those under three months of age—remain disproportionately affected, as they are either unvaccinated or only partially immunized. Data from the Centers for Disease Control and Prevention (CDC) indicate that nearly half of pertussis‐related hospitalizations and the vast majority of deaths occur in this age group [11], with similar findings reported in large population‐based studies [2]. These observations reinforce the narrow window available for intervention in this population and the need for early recognition of disease severity.

**FIGURE 1:**
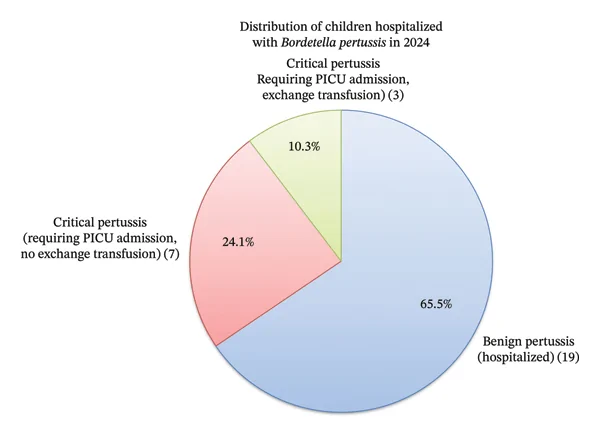
Distribution of children hospitalized with *Bordetella pertussis* in 2024.

During the study period, 29 children were admitted with pertussis, of whom 10 required PICU care for critical disease. Three patients met predefined criteria for exchange transfusion. While not the primary focus of this case report, these data provide important context regarding institutional disease burden and the proportion of patients progressing to life‐threatening illness. The distribution of hospitalized children according to disease severity and exchange transfusion status is shown in Figure 1.

The infants included in our report were young and largely within the highest‐risk age group, including those under three months of age, and were unvaccinated with a rapidly progressive clinical course‐findings that are consistent with previously described patterns of critical pertussis. Notably, maternal Tdap vaccination was not part of routine antenatal care in Kuwait prior to January 2025. This gap likely contributed to early infant vulnerability, as maternal immunization is well established to confer passive protection during the highest‐risk period [12, 13]. Tdap vaccination during each pregnancy, optimally between 27 and 36 weeks of gestation, allows adequate maternal antibody generation and transplacental transfer, helping protect the infant during the first months of life before adequate protection from the infant’s own immunization series [13, 14]. This underscores an important and modifiable public health opportunity.

Our findings contribute to a growing body of literature supporting early exchange transfusion as a potentially life‐saving intervention in critical pertussis [1, 4–6]. Hyperleukocytosis has been consistently associated with pulmonary vascular obstruction and the development of pulmonary hypertension, which remains a primary driver of mortality [1, 2, 6]. Observational studies have suggested that early leukoreduction—particularly when performed prior to the onset of refractory pulmonary hypertension—may improve outcomes [4, 5]. Although no universally accepted leukocyte threshold exists, many centers adopt pragmatic triggers based on white blood cell count together with clinical deterioration, particularly in young infants [1, 4–6].

In this context, our anticipatory, consensus‐based approach—integrating early laboratory and clinical triggers—allowed for timely intervention prior to irreversible cardiopulmonary compromise. Across all three cases, exchange transfusion performed within 2–4 days of admission resulted in consistent leukoreduction and rapid clinical stabilization. Case 1 required a second exchange transfusion after the WBC count rebounded to approximately 25 × 10^9^/L, accompanied by persistent/worsening clinical findings. Thrombocytopenia following exchange transfusion was transient and manageable. Seizures and iatrogenic opioid withdrawal in Cases 1 and 2 occurred during the broader PICU course and were not considered complications directly attributable to exchange transfusion. The absence of mortality and favorable short‐term recovery in all patients further supports the potential benefit of early intervention.

Beyond individual patient outcomes, this case report highlights important system‐level considerations. Postintervention feedback from PICU leaders across Kuwait demonstrated strong support for the development of a standardized national protocol. However, variability in practice—particularly in relation to blood product preparation, exchange techniques, and trigger thresholds—remains a significant barrier to consistent care delivery. This heterogeneity reflects the absence of unified guidance and underscores the need for coordinated national strategies. The findings of the national needs assessment across seven PICUs are summarized in Table 2.

**TABLE 2:** Needs’ assessment for exchange transfusion protocol across 7 PICUs in Kuwait.

Response	Number of PICUs
Yes	7
No	0

Despite encouraging findings, the broader implementation of exchange transfusion remains limited by logistical challenges, including resource availability, technical expertise, and coordination of multidisciplinary teams. Addressing these barriers will be essential to ensure equitable access and optimize outcomes. Taken together, our findings support a shift toward earlier, structured, and system‐supported intervention strategies in the management of critical pertussis.

## 5. Conclusion

Early exchange transfusion appears safe and potentially life‐saving in infants with critical pertussis, particularly when initiated before refractory pulmonary hypertension develops. Our findings support proactive intervention guided by combined clinical and laboratory triggers.

These findings also support the need for structured implementation strategies, including early identification protocols, standardized exchange transfusion practices, maternal Tdap immunization, and strengthened national surveillance systems to reduce mortality in this vulnerable population.

## Funding

No funding was received for this manuscript.

## Disclosure

This case report was prepared in accordance with the CARE reporting guideline.

## Ethics Statement

Formal ethical approval was not required for this case report in accordance with institutional requirements, as it describes individual cases and their clinical management without any research‐specific intervention. Patient confidentiality was maintained throughout.

## Consent

Written informed consent for publication of the clinical details was obtained from the parents/legal guardians of all three patients.

## Conflicts of Interest

The authors declare no conflicts of interest.

## Supporting Information

Additional supporting information can be found online in the Supporting Information section.

## Supporting information


**Supporting Information** CARE Checklist.

## Data Availability

The data that support the findings of this study are available in the supporting information of this article.
