# Supplementary material for: Early Exchange Transfusion in Critical Pertussis: A Case Report Supporting Timely Intervention
Source: Case Rep Pediatr. 2026 Sep 4;2026:7016973. doi: 10.1155/crpe/7016973 (PMC13542994; doi:10.1155/crpe/7016973)
Supplement: Supplementary file 1 [file CRPE-2026-7016973-s001.docx]

**2013 CARE Checklist – Completed for Submission**

**Manuscript: Early Exchange Transfusion in Critical Pertussis: A Case Report Supporting Timely Intervention**

The checklist below reproduces the full 13-item CARE structure and maps each sub-item to the revised manuscript. Items not supported by the source manuscript are identified rather than inferred.

| **Item** | **Topic** | **CARE checklist item** | **Location in manuscript** | **Reported?** |
| --- | --- | --- | --- | --- |
| 1 | Title | The diagnosis or intervention of primary focus followed by the words “case report”. | Title | Yes |
| 2 | Key Words | 2 to 5 key words that identify diagnoses or interventions in this case report (including “case report”). | Not currently included in manuscript | Not reported |
| 3a | Abstract – Introduction | What is unique about this case and what does it add to the scientific literature? | Abstract – Background/Objectives | Yes |
| 3b | Abstract – Clinical findings | The patient’s main concerns and important clinical findings. | Abstract – Results | Yes |
| 3c | Abstract – Diagnosis/intervention/outcomes | The primary diagnoses, interventions, and outcomes. | Abstract – Methods/Results | Yes |
| 3d | Abstract – Conclusion | One or more take-away lessons from the case report. | Abstract – Conclusion | Yes |
| 4 | Introduction | Briefly summarizes why this case is unique and may include medical literature references. | Introduction | Yes |
| 5a | Patient Information | De-identified patient-specific information. | Cases 1–3 | Yes |
| 5b | Patient Information | Primary concerns and symptoms of the patient. | Cases 1–3 | Yes |
| 5c | Patient Information | Medical, family, and psychosocial history including relevant genetic information. | Cases 1–3 | Reported |
| 5d | Patient Information | Relevant past interventions and their outcomes. | Cases 1–3 | Yes, where applicable |
| 6 | Clinical Findings | Describe significant physical examination and important clinical findings. | Cases 1–3 | Reported |
| 7 | Timeline | Historical and current information from this episode of care organized as a timeline (figure or table). | Cases 1–3 and Table 1 | Reported |
| 8a | Diagnostic Assessment | Diagnostic methods (physical examination, laboratory testing, imaging, surveys). | Methods and Cases 1–3 | Yes – PCR, laboratory trends and echocardiography reported |
| 8b | Diagnostic Assessment | Diagnostic challenges. | — | Not reported |
| 8c | Diagnostic Assessment | Diagnosis, including other diagnoses considered. | Methods and Cases 1–3 | Reported |
| 8d | Diagnostic Assessment | Prognostic characteristics when applicable. | Cases 1–3 and Discussion | Yes |
| 9a | Therapeutic Intervention | Types of therapeutic intervention (pharmacologic, surgical, preventive). | Cases 1–3 and Table 1 | Yes |
| 9b | Therapeutic Intervention | Administration of therapeutic intervention (dosage, strength, duration). | Methods, Cases 1–3 and Table 1 | Reported |
| 9c | Therapeutic Intervention | Changes in therapeutic interventions with explanations. | Case 1 and Discussion | Yes – second exchange explained by rebound WBC with persistent/worsening clinical findings |
| 10a | Follow-up and Outcomes | Clinician- and patient-assessed outcomes, if available. | Cases 1–3 | Yes – clinician-assessed outcomes reported |
| 10b | Follow-up and Outcomes | Important follow-up diagnostic and other test results. | Cases 1–3 | Reported |
| 10c | Follow-up and Outcomes | Intervention adherence and tolerability, and how assessed. | N/A | N/A |
| 10d | Follow-up and Outcomes | Adverse and unanticipated events. | Abstract, Cases 1–2, Discussion and Table 1 note | Yes – thrombocytopenia distinguished from seizures and iatrogenic opioid withdrawal during broader PICU course |
| 11a | Discussion | Strengths and limitations in the approach to this case. | Discussion | Reported |
| 11b | Discussion | Discussion of the relevant medical literature. | Discussion | Yes |
| 11c | Discussion | Rationale for conclusions. | Discussion | Yes |
| 11d | Discussion | Primary take-away lessons from this case report in a one-paragraph conclusion without references. | Conclusion | Yes |
| 12 | Patient Perspective | The patient should share their perspective on the treatment(s) they received. | N/A | N/A |
| 13 | Informed Consent | The patient should give informed consent (provide if requested). | Written Informed Consent statement | Reported |
